# Giant calyceal diverticulum diagnosed in pregnancy: A case report of successful percutaneous catheter management during pregnancy and robot-assisted calyceal diverticulectomy after delivery

**DOI:** 10.1016/j.eucr.2025.102935

**Published:** 2025-01-09

**Authors:** Takashi Sakaguchi, Yoichi Osako, Akihiko Mitsuke, Risako Ogawa, Himawari Takeyama, Ryosuke Matsushita, Hirofumi Yoshino, Shuichi Tatarano, Hideki Enokida

**Affiliations:** Department of Urology, Graduate School of Medical and Dental Sciences, Kagoshima University, Kagoshima, Japan

**Keywords:** Renal pelvis, Diverticulum, Percutaneous catheter, Robot-assisted surgery, Pregnancy

## Abstract

We report our experience with a pregnant patient with a giant calyceal diverticulum—a very rare presentation. A 30-year-old pregnant woman was referred to our department with left flank pain at 7 weeks of gestation. Ultrasonography and computed tomography showed a giant cystic lesion on the left kidney. Single-puncture drainage was performed, but when fluid immediately reaccumulated, a percutaneous catheter was placed. We suspected a fistula between the cystic lesion and renal pelvis. After delivery, we made a definitive diagnosis of a calyceal diverticulum and successfully performed robot-assisted calyceal diverticulectomy.

## Highlights

• ・This case reports a rare instance of huge calyceal diverticulum in a pregnant woman.
• ・She was appropriately managed during pregnancy with a percutaneous catheter.
• ・After delivery, she underwent curative robot-assisted surgery.
• ・There is no evidence of recurrence 5 months after surgery.

## 1 Introduction

Calyceal diverticulum (CD) is a rare congenital malformation of the urinary tract, consisting of a cystic lesion that communicates with the renal pelvis and having an incidence of approximately 0.21%–0.4 %.^1^^,^^2^ When it is symptomatic, aggressive therapeutic intervention may be required.^3^ Herein, we report our experience with a patient who had a very rare presentation: a giant CD diagnosed during pregnancy. She was managed appropriately with a percutaneous catheter during pregnancy and definitively treated with robotic surgery after delivery with a good outcome.

## 2 Case presentation

**Fig. 1:**
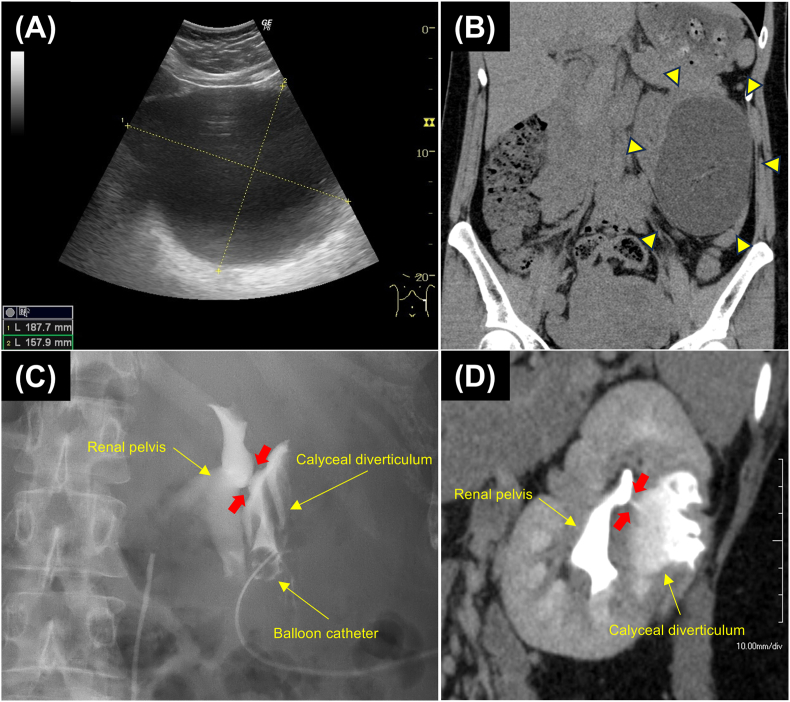
Composite preoperative images (A) Abdominal ultrasonography and (B) computed tomography (CT) during pregnancy demonstrates a huge cystic lesion (B, arrowheads) on the outer side of the left kidney. (C) Retrograde pyelography and (D) contrast-enhanced CT after delivery reveal a calyceal diverticulum with a small fistula (arrows) communicating with the renal pelvis.

A 30-year-old nulliparous woman at 7 weeks of gestation was referred to our department with left flank pain, abdominal distention, and anorexia. About 2 years prior, an asymptomatic cystic lesion of the left kidney, about 5 cm in size, was noted at another hospital but was not evaluated further. Ultrasonography revealed a cystic lesion measuring 18 × 15 cm on the left kidney, which was thought to be a simple renal cyst (Fig. 1A). We performed single-puncture drainage and removed 1500 mL of yellowish clear fluid. However, only a few days later, the patient reported severe abdominal distension, and ultrasonography revealed fluid reaccumulation to the same volume as before the puncture.

We placed a 6-Fr pigtail catheter into the lesion and performed plain computed tomography (CT), which revealed findings consistent with a simple renal cyst (Fig. 1B). However, assessment of the drained fluid revealed a high creatinine level (29.37 mg/dL), and a urinary fistula was suspected. Because of the potential impact on the fetus, the plan was to avoid iodinated contrast imaging and to continue percutaneous catheter management until delivery. Eventually, the catheter was changed to a 10-Fr balloon catheter. To accomplish this, we injected approximately 100 mL of saline through the 6-Fr pigtail catheter into the diverticulum to allow visualization by abdominal ultrasonography. A guidewire was inserted, a dilator was used to expand the tract, and the original catheter was replaced with a 10-Fr balloon catheter. The guidewire, dilator, and balloon catheter were all well visualized and were safely exchanged without the need for contrast imaging. The balloon catheter was changed every 3 weeks using the same technique, allowing for safe management until delivery. The patient underwent successful vaginal delivery at 37 weeks and 1 day of gestation.

**Fig. 2:**
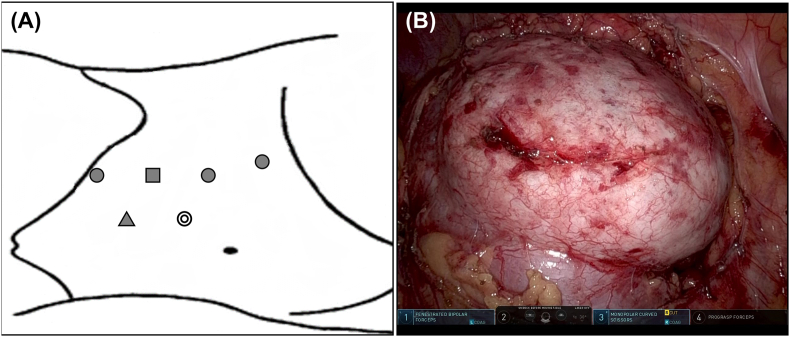
Port placement and appearance of calyceal diverticulum (A) Schematic of port placement: circles, 8-mm robotic ports; square, 8-mm camera port; triangle, 5-mm assistant port; double circle, 12-mm assistant port; distance between each port is 7 cm. (B) Calyceal diverticulum.

**Fig. 3:**
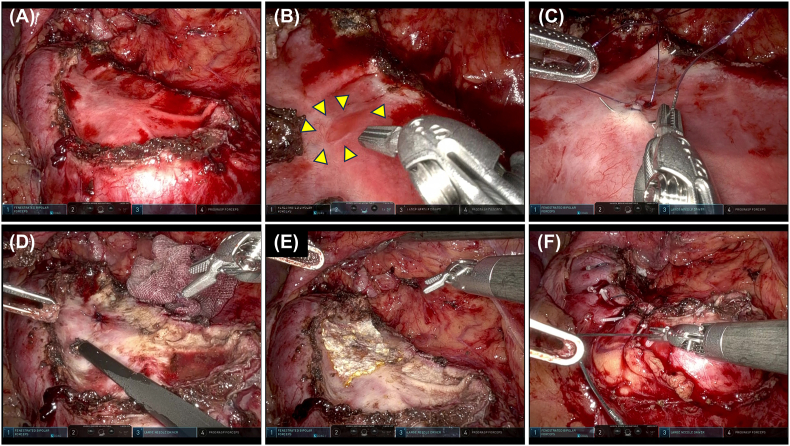
Surgical precedure (A) Resection of the diverticular wall and identification of the fistula. (B) Proximal view of fistula (arrowheads). (C) Closure of the fistula with continuous suturing. (D) Cautery of the diverticular lumen using monopolar soft coagulation. (E) The fistula closure is sealed using a tissue adhesive sheet. (F) Renorrhaphy with sliding clip technique.

After delivery, the catheter continued to drain urine at a rate of more than 1000 mL/day. Contrast-enhanced CT and retrograde pyelography revealed a cystic lesion communicating with the renal calyx through a small fistula (Fig. 1C and D). At this point, we diagnosed a CD and performed robot-assisted calyceal diverticulectomy using the da Vinci Xi surgical system (Intuitive Surgical, Sunnyvale, CA, USA). The port placement is shown in Fig. 2A. Excision of the diverticular wall revealed a fistula that appeared to be a diverticular orifice (Fig. 2, Fig. 3A, B). The fistula was closed by continuous suturing using 4-0 poliglecaprone (Monocryl) in a watertight fashion (Fig. 3C). The diverticular lumen was cauterized using monopolar soft coagulation, and the fistula closure was sealed using a tissue adhesive sheet (TachoSil; CSL Behring Japan, Tokyo, Japan) (Fig. 3D and E). Finally, we performed renorrhaphy with the sliding-clip technique using a V-Loc wound closure device with a 2-0 suture (Covidien™, Mansfield, MA, USA) (Fig. 3F). A 6-Fr double-J ureteral stent was placed immediately after surgery and removed 8 weeks after surgery.

**Fig. 4:**
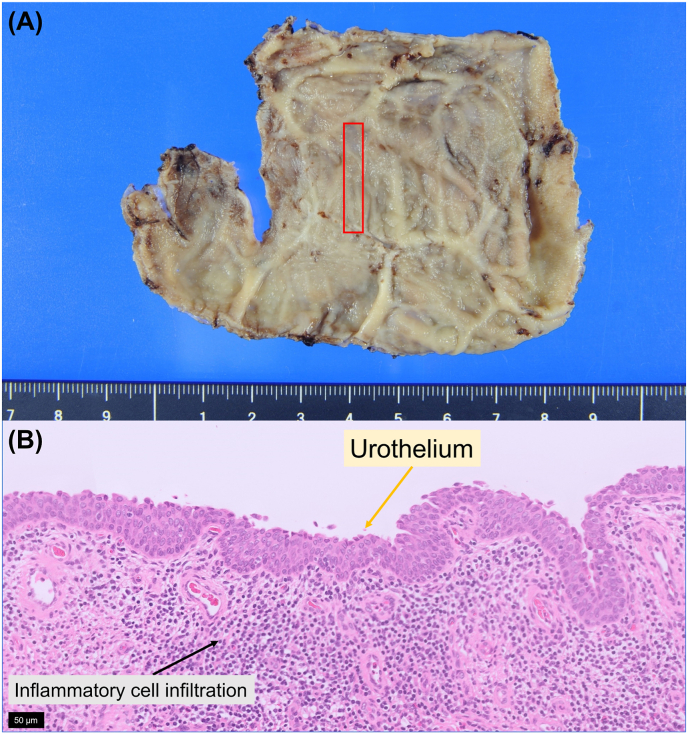
Histopathologic findings (A) Gross view of the cyst wall. (B) Microscopy (hematoxylin and eosin staining) shows a cyst wall covered with erosive inflamed urothelium, consistent with a calyceal diverticulum.

**Fig. 5:**
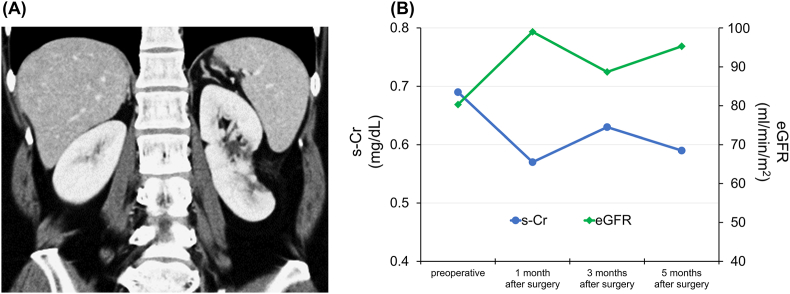
Postoperative findings (A) Contrast enhanced CT shows no evidence of recurrence of the calyceal diverticulum 5 months after surgery. (B) Renal function over time. s-Cr, serum creatinine; eGFR, estimated glomerular filtration rate.

The patient had an uneventful perioperative course. Histologic examination revealed a cyst wall covered with urothelium, confirming the final diagnosis of CD (Fig. 4). There has been no evidence of diverticulum recurrence on CT 5 months after surgery and no deterioration of renal function (Fig. 5).

## 3 Discussion

Most CDs are asymptomatic, but these lesions can cause diverticular stones (10%–40 %), urinary tract infection (25 %), and pain (25 %).^4^ There are only 3 reports of symptoms appearing during pregnancy leading to diagnosis and treatment; our patient represents an extremely rare presentation.^5^, ^6^, ^7^ In general, increased intrarenal pelvic pressure during pregnancy can be attributed to physical compression by the gravid uterus or to decreased ureteral tone and peristalsis due to the effects of progesterone and human chorionic gonadotropin 8. We speculate that the latter may be one of the causes of our patient's increased intrarenal pelvic pressure, given that she was in the early stages of pregnancy at presentation.

Determining whether the cystic lesion communicates with the renal collecting system is important in distinguishing CD from simple renal cysts; contrast radiography is useful for this purpose.^9^ There have been reports of misdiagnosis of CD as a simple renal cyst, resulting in subsequent treatment difficulties.^10^ However, contrast radiography is contraindicated during pregnancy because of the potential fetal side effects.^5^ For this reason, we did not perform radiographic examinations using contrast agents during pregnancy, but we were able to consider the possibility of a CD because of the nature of the contents of the cystic lesion, ie, consistent with urine. We were able to make the definitive diagnosis of CD with postpartum radiographic examination using contrast.

Surgical treatment, such as stone removal, excision, or cauterization of diverticular walls, is indicated when CD manifests as recurrent urinary tract infection, pain, or hematuria.^4^^,^^11^^,^^12^ In addition to urologic endoscopic surgery, several recent reports have demonstrated the safety and utility of laparoscopic and robotic surgery.^13^, ^14^, ^15^, ^16^ Taylor et al. reported on 6 patients undergoing minimally invasive calyceal diverticulectomy (4 laparoscopic and 2 robotic); the authors noted that symptoms resolved in all patients with no serious complications.^13^ Gonzalez et al. report that percutaneous and ureteroscopic treatment results in diverticular resolution rates of 60 % and 20 %, respectively, while laparoscopic diverticulectomy has a success rate as high as 90 %–100 %.^14^ There are no clear criteria for choosing a surgical modality for CD, but several techniques have been mentioned in previous reports.^3^^,^^4^^,^^9^^,^^12^ The percutaneous endoscopic approach is appropriate for diverticula located posterior to the kidney at the mid portion or lower pole, but it is inappropriate for diverticula located anterior to the kidney.^4^^,^^9^ The ureteroscopic approach is minimally invasive and is appropriate for diverticula located anterior to the kidney at the upper pole, but it is inappropriate for diverticula located at the lower pole or when access to the diverticulum is at a sharp angle.^4^^,^^9^^,^^12^ It has also been reported that the diverticular orifice cannot be identified by ureteroscopy in 30 % of patients, which is a major concern.^4^ Laparoscopic or robot-assisted surgery is expected to have the best outcome in terms of reliable resection of the diverticular wall and closure of the diverticular orifice, but there are concerns about increased surgical invasiveness when the renal parenchyma surrounds the diverticulum.^3^ However, when the renal parenchyma surrounding the diverticulum is thin and when there is with a large exophytic component to the CD, these approaches are certainly indicated.^3^^,^^12^ The choice of treatment approach for patients with CD should be comprehensively evaluated according to the anatomic location and size of the diverticulum and the status of its orifice. Currently, there is no clear evidence for the superiority of laparoscopic vs robotic surgery for calyceal diverticulectomy. Taylor et al. found that laparoscopic surgery is good for thin-walled, anteriorly located diverticula, while robot-assisted surgery is preferred when clamping or renorrhaphy is required, when there are large diverticula with stones, or for posteriorly located diverticula.^13^ They also note that 3-dimensional visualization and the ability to perform complex reconstructions are major advantages of robot-assisted calyceal diverticulectomy.^13^ In addition, Akca et al. report that laparoscopic CD resection may take longer and present more difficulties with suture manipulation than robot-assisted surgery.^17^ In our patient, the choice of robot-assisted surgery was appropriate because the diverticulum was huge, although not complicated by stones, and required renorrhaphy. In addition, we were able to take advantage of reliable visualization and to completely close the diverticular orifice using suture. We believe that a cure was achieved for our patient without major complications due to the reliable resection of the diverticulum and precise suturing technique allowed by robotic surgery.

## 4 Conclusion

Appropriate percutaneous catheter management during pregnancy and robot-assisted surgery after delivery may be effective treatment options for giant renal CD diagnosed during pregnancy.

## CRediT authorship contribution statement

**Takashi Sakaguchi:** Conceptualization, Writing – original draft. **Yoichi Osako:** Writing – review & editing. **Akihiko Mitsuke:** Validation. **Risako Ogawa:** Validation. **Himawari Takeyama:** Validation. **Ryosuke Matsushita:** Validation. **Hirofumi Yoshino:** Validation. **Shuichi Tatarano:** Writing – review & editing. **Hideki Enokida:** Writing – review & editing.

## Consent

Written informed consent for publication was obtained from the patient.

## Funding

This research did not receive any specific grant from funding agencies in the public, commercial or not-for-profit sectors.

## Declaration of competing interest

The authors declare that they have no competing interests.
